# Fibrillar beta-amyloid peptide Aβ_1–40 _activates microglial proliferation via stimulating TNF-α release and H_2_O_2 _derived from NADPH oxidase: a cell culture study

**DOI:** 10.1186/1742-2094-3-24

**Published:** 2006-09-07

**Authors:** Aiste Jekabsone, Palwinder K Mander, Anna Tickler, Martyn Sharpe, Guy C Brown

**Affiliations:** 1 Department of Biochemistry, University of Cambridge, Tennis Court Road, Cambridge CB2 1QW, UK; 2 Cavendish laboratory, University of Cambridge, Cambridge CB3 0HE, UK; 3 Biochemistry and Molecular Biology Department, Biochemistry Building, Michigan State University, East Lansing, MI 48824-1319, USA

## Abstract

**Background:**

Alzheimer's disease is characterized by the accumulation of neuritic plaques, containing activated microglia and β-amyloid peptides (Aβ). Fibrillar Aβ can activate microglia, resulting in production of toxic and inflammatory mediators like hydrogen peroxide, nitric oxide, and cytokines. We have recently found that microglial proliferation is regulated by hydrogen peroxide derived from NADPH oxidase. Thus, in this study, we investigated whether Aβ can stimulate microglial proliferation and cytokine production via activation of NADPH oxidase to produce hydrogen peroxide.

**Methods:**

Primary mixed glial cultures were prepared from the cerebral cortices of 7-day-old Wistar rats. At confluency, microglial cells were isolated by tapping, replated, and treated either with or without Aβ. Hydrogen peroxide production by cells was measured with Amplex Red and peroxidase. Microglial proliferation was assessed under a microscope 0, 24 and 48 hours after plating. TNF-α and IL-1β levels in the culture medium were assessed by ELISA.

**Results:**

We found that 1 μM fibrillar (but not soluble) Aβ_1–40 _peptide induced microglial proliferation and caused release of hydrogen peroxide, TNF-α and IL-1β from microglial cells. Proliferation was prevented by the NADPH oxidase inhibitor apocynin (10 μM), by the hydrogen peroxide-degrading enzyme catalase (60 U/ml), and by its mimetics EUK-8 and EUK-134 (20 μM); as well as by an antibody against TNF-α and by a soluble TNF receptor inhibitor. Production of TNF-α and IL-1β, measured after 24 hours of Aβ treatment, was also prevented by apocynin, catalase and EUKs, but the early release (measured after 1 hour of Aβ treatment) of TNF-α was insensitive to apocynin or catalase.

**Conclusion:**

These results indicate that Aβ_1–40_-induced microglial proliferation is mediated both by microglial release of TNF-α and production of hydrogen peroxide from NADPH oxidase. This suggests that TNF-α and NADPH oxidase, and its products, are potential targets to prevent Aβ-induced inflammatory neurodegeneration.

## Background

Alzheimer's disease is characterised by neuritic plaques that contain dead and dying neurons and their processes, inflammatory-activated microglia and β-amyloid peptides Aβ_1–40 _and Aβ_1–42 _[1,2]. The disease is accompanied by brain inflammation, characterised by increased cytokine levels and increased numbers of activated microglia [3]. Epidemiological studies have indicated that non-steroidal anti-inflammatory drugs (cyclooxygenase inhibitors) prevent or delay the onset of Alzheimer's, suggesting that brain inflammation contributes to disease progression prior to clinical symptoms [4,5]. β-Amyloid and cytokines cause inflammatory activation of glia, and inflammatory-activated microglia are consistently found in the neuritic plaques of Alzheimer's patients [1,2]. β-Amyloid, cytokines and/or bacteria-activated microglia potently kill co-cultured neurons, and the ultimate means by which neurons are killed in a wide range of brain pathologies may be inflammatory neurodegeneration mediated by activated microglia [3,6-9]. It is therefore vital to understand how glial activation and subsequent neuronal death can be prevented.

Microglia have a specific NADPH oxidase known as PHOX (phagocytic oxidase), consisting of subunits gp91 (NOX2), p22, p47, p67, p40 and Rac [3]. Normally in resting microglia this oxidase is relatively inactive and unassembled, but when activated by β-amyloid, bacteria and/or cytokines, the oxidase assembles at the plasma membrane, and produces superoxide that is released extracellularly or into phagosomes at a high rate. The superoxide either dismutates to hydrogen peroxide or reacts with nitric oxide to produce cytotoxic peroxynitrite [7,8]. We and others believe that NADPH oxidase activation is the key event converting resting microglia to activated, proliferating, cytotoxic microglia; and, therefore, that blocking oxidase activation may block inflammatory neurodegeneration [3,6,8-12].

We have recently found that proliferation of microglia is dependent on H_2_O_2 _from PHOX, that cytokines, arachidonate and ATP stimulate microglial proliferation via stimulating H_2_O_2 _production from PHOX; and that inhibiting PHOX prevents this [10]. We also found that microglial PHOX and reactive oxygen and nitrogen species are key mediators of inflammatory killing of neurons [7,8,13-15]. Others have shown that activation of H_2_O_2 _production from PHOX is a required step for inflammatory activation of microglia (measured by iNOS expression and cytokine production) induced by LPS [11,12]. Since β-amyloid is known to activate superoxide or H_2_O_2 _production from the microglial PHOX [9,16,17], we test here whether this activation is responsible for β-amyloid-induced proliferation of microglia and cytokine production.

## Materials and methods

### Materials

Apocynin was purchased from Calbiochem; EUK-8 and EUK-134 were synthesized as previously described in [18]; Amplex Red, Dulbecco's Modified Eagle Medium (DMEM), Earl's Balanced Salt Solution (EBSS), Isolectin GS-IB4 from *Griffonia simplicifolia *conjugated with AlexaFluor488, Phosphate-Buffered Saline (PBS), ready-to-use Streptavidin-horse radish peroxidase (HRP) conjugate were purchased from Invitrogen; Anti-rat TNF-α monoclonal antibody was purchased from R&D Systems; soluble TNF receptor inhibitor/Fc chimera was purchased from GenScript Corporation; Biotin anti-rat TNF-α polyclonal antibody was purchased from Insight Biotechnology Ltd.; all other chemicals were purchased from Sigma.

### Aβ_1–40 _peptide source, fibrillization, and identification of fibrillization state

Aβ_1–40 _peptide was synthesized as described [19]. The peptide was dissolved in water to make a stock solution of 0.1 mM. Part of the solution (further used as soluble Aβ_1–40 _peptide stock solution) was immediately aliquoted in small single-use fractions and stored at -20°C. Another part of the solution was kept at 37°C for 7 days to induce peptide aggregation and fibril formation. Fibrillization of the peptide was confirmed by its ability to change Thioflavine T fluorescence spectra, as described [20]. A 5 μM solution of fibrillar peptide in PBS (pH6.0) changed Thioflavine T (3 μM) fluorescence spectra: in excitation spectrum it induced a peak at λ_em _= 450 nm, and in emission spectrum a peak appeared at λ_ex _= 482 nm. None of these peaks could be seen with the dye alone or with the dye plus 5 μM soluble Aβ_1–40 _peptide. After aggregation, the stock solution of fibrillar Aβ_1–40 _peptide was also aliquoted in smaller fractions and stored at -20°C. After re-thawing the fibrillar state of the peptide remained unchanged.

### Preparation of pure microglial culture

Primary mixed astrocyte and microglial cultures were used for pure microglial culture preparation. Mixed glial cultures were prepared from the cerebral cortices of 7-day-old Wistar rats. After dissection of the cerebral hemispheres, meninges were removed and the tissue was dissociated in a solution of EBSS containing 0.3% BSA, 103.2 Kunitz units/ml DNase I and 3800 BAEE units/ml Trypsin. Cells were plated at 2 × 10^5 ^cells/cm^2 ^in 75 cm^2 ^flasks coated with 0.0005% poly-L-lysine. Cultures were maintained in DMEM supplemented with 10% foetal calf serum and 1 mg/ml gentamicin. Cells were kept at 37°C in a humidified atmosphere of 5% CO_2 _and 95% air.

When mixed glial cultures reached confluency (on 6^th^–8^th ^day *in vitro*), microglial cells were isolated by shaking and tapping the flasks. Medium from the mixed glial cultures, containing dislodged microglial cells, was removed and centrifuged (135 g) for 5 min. The supernatant was discarded and cells were resuspended either in DMEM with the same supplements as for mixed glial cultures (for proliferation and inflammatory cytokines measurements) or in Hanks' Balanced Salt Solution (for hydrogen peroxide assay).

### Assessment of microglial culture purity, cell viability, and proliferation

After isolation from mixed glial cultures, microglial cells were plated in 96-well plates at 15 × 10^3 ^cells/cm^2^. Two hours after plating, cultures were stained with isolectin IB_4_-AlexaFluor488 conjugate, which has a strong affinity for microglia but not for astrocytes [21]. The dye specifically stains microglia regardless of their activation status [22], and we found that it does not affect cell viability or proliferation up to 72 hours; thus, it can be used for visualization of microglia before the start of experiment. Isolectin IB_4_, 10 ng/ml, was added to cells and incubated for 15 min at 37°C. Stained cells were counted using fluorescence microscope Axiovert S-100 (λ_ex _= 488 nm, λ_em _= 530 nm), and all cells were counted under light phase contrast in the same microscopic fields. The purity of the cultures was 99.70 ± 0.01% (mean ± SE, n = 12). The number of IB_4_-stained cells per microscopic field was considered as cell number at time '0'. Then, treatment was started with 1 μM fibrillar Aβ_1–40 _or 1 μM fibrillar Aβ_1–40_, alone or together with either (*i*) 10 μM apocynin (an NADPH oxidase inhibitor), (*ii*) 60 IU/ml catalase (the enzyme that converts hydrogen peroxide to water and oxygen), (*iii*) either of the catalase mimetics EUK-8 or EUK-134 (both at 20 μM), (*iv*) 40 μg/ml anti-TNF-α, or (*v*) 10 ng/ml soluble TNF receptor inhibitor. Experiments were also performed with 1 μM soluble Aβ_1–40_, with apocynin, catalase or EUKs without Aβ; with 10 pg/ml phorbol 12-myristate 13-acetate (PMA, an NADPH oxidase activator), or with PMA together with apocynin or catalase.

After 24 or 48 hours, the cultures were stained again with isolectin IB_4 _for detection of microglia (as described above), with Hoechst 33342 for visualization of all nuclei and for detection of chromatin-condensed (apoptotic) nuclei, and with propidium iodide for detection of necrotic nuclei (10 μg/ml Hoechst, 2 μg/ml propidium iodide, incubated 5 min at room temperature, visualized using fluorescence microscope at λ_ex _= 365 nm, λ_em _= 420 nm).

In every experiment, cells were counted in 5 microscopic fields for each well, and there were 6 wells for each treatment, as well as for untreated cells. The total number of cells counted for each treatment was 314 – 875.

There were no chromatin-condensed nuclei detected in the cultures. The percentage of necrotic microglial nuclei was 0.75 ± 0.03 (mean ± SE, n = 7) after 24 hours, and 1.54 ± 0.07 (n = 5) after 48 hours, and this did not differ significantly between untreated and amyloid β peptide-, apocynin-, catalase- or EUKs-treated cultures. Microglial numbers after 24 and 48 hours of incubation were expressed as percentage of time '0' numbers, and this was considered as a measure of microglial proliferation.

### Measurement of TNF-α and IL-1β concentration

Pure microglial cultures were incubated under the same conditions and with the same treatments as for proliferation measurements (described above), except for anti-TNF-α treatment. The culture medium was collected from cells after 1, 6, 24 or 48 hours. The amounts of inflammatory cytokines in the medium were detected by ELISA. The data presented in Fig. 4 and 5 are obtained by using kits for rat TNF-α and IL-1β (Quantikine, R&D Systems), assaying the samples according to the manufacturer's protocol provided with the kits. For the data in Fig. 6 and 7 the following protocol was used: clear polystyrene microplates (R&D Systems) were covered with monoclonal anti-rat TNF-α antibody (100 μl/well, 20 μg/ml) by incubating overnight at room temperature. Then the wells were aspirated and washed with Wash buffer (0.05% Tween 20 in PBS, pH7.4) 5 times (the aspiration and washing with the same buffer was repeated before each following addition), and the plates were blocked with 300 μl per well of blocking solution (PBS containing 1% BSA and 5% sucrose) for 2 hours at room temperature. Then, 50 μl of the blocking solution was added to each well followed by the addition of 100 μl per well of samples or standards diluted in PBS (the standards from TNF-α ELISA Kit, R&D Systems, were used) and the plates were incubated 2 hours at room temperature. After this, 100 μl of biotin anti-TNF (2 μg/ml of the blocking solution) was added to each well and incubated for 2 hours at room temperature, followed by a 20 min incubation with 100 μl (2 drops) per well of streptavidin-HRP ready-to use solution. Finally, 100 μl/well substrate solution (0.05% 3,3',5,5'-tetramethylbenzidine and 0.012% H_2_O_2 _in 0.05 M citrate buffer, pH5.0) was added and incubated for 30 minutes at 37°C. The reaction was stopped with 1 M H_2_SO_4_, 50 μl/well, and the optical density at λ = 450 nm was measured in a microplate reader (Emax, Molecular Devices). Concentrations of TNF-α in the samples were calculated from the calibration curve constructed using known amounts of rat TNF-α standards.

**Figure 1:**
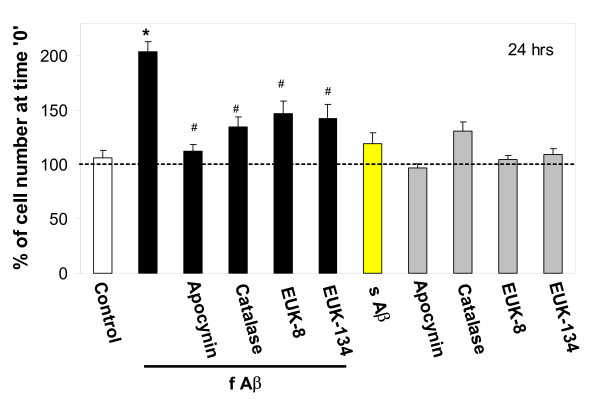
The effect of Aβ_1–40 _peptide on microglial proliferation at 24 hours. Microglial cultures were incubated with 1 μM fibrillar (f) or soluble (s) Aβ_1–40_, the NADPH oxidase inhibitor apocynin (10 μM), and/or with the hydrogen peroxide converters catalase (60 IU/ml) or EUK-8/-134 (20 μM) for 24 hours. After a 24 hour incubation, microglial cells were counted and their numbers expressed as percentage of cell number at time '0'. The dashed line indicates cell number at time '0' i.e. 100%. Data are expressed as mean of 5 experiments ± standard error. Statistical analysis used: Student's t-test, p < 0.05; * – significant difference compared to the control; # – significant difference compared to samples treated with fibrillar Aβ only.

**Figure 2:**
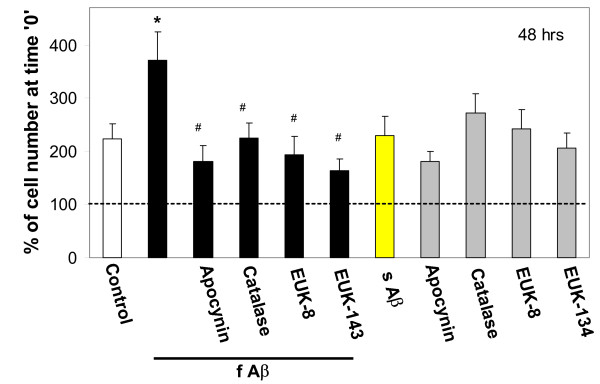
The effect of Aβ_1–40 _peptide on microglial proliferation at 48 hours. Microglial cultures were incubated with 1 μM fibrillar (f) or soluble (s) Aβ_1–40_, the NADPH oxidase inhibitor apocynin (10 μM), and/or with the hydrogen peroxide converters catalase (60 IU/ml) and EUK-8/-134 (20 μM) for 48 hours. After a 48 hour incubation, microglial cells were counted and their numbers expressed as percentage of cell number at time '0'. The dashed line indicates cell number at time '0'. Data are expressed as mean of 5 experiments ± standard error. Statistical analysis used: Student's t-test, p < 0.05; * – significant difference compared to the control; # – significant difference compared to samples treated with fibrillar Aβ only.

**Figure 3:**
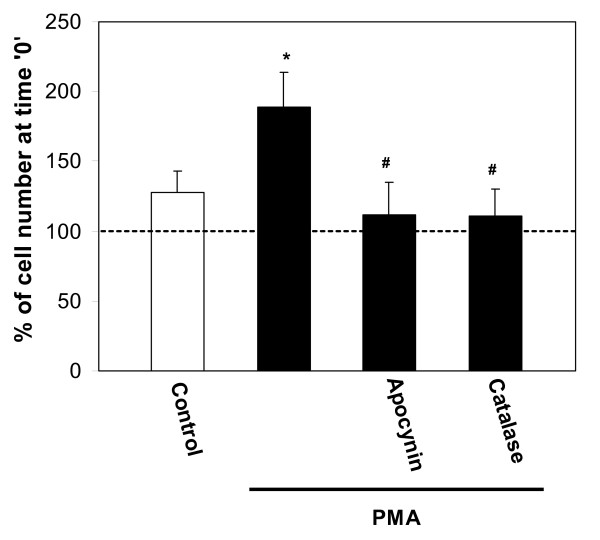
Microglial proliferation stimulated by PMA. Microglial cultures were incubated with 10 pg/ml of the NADPH oxidase activator phorbol 12-myristate 13-acetate (PMA), alone or together with the NADPH oxidase inhibitor apocynin (10 μM), or the hydrogen peroxide converter catalase (60 IU/ml) for 24 hours; then microglial cells were counted and their numbers were expressed as percentage of cell number at the start of the treatment, or time'0'. The dashed line indicates cell number at time '0'. Data are expressed as mean of 4 experiments ± standard error, Statistical analysis used: Student's t-test, p < 0.05; * – significant difference compared to the control; # – significant difference compared to samples treated with PMA only.

**Figure 4:**
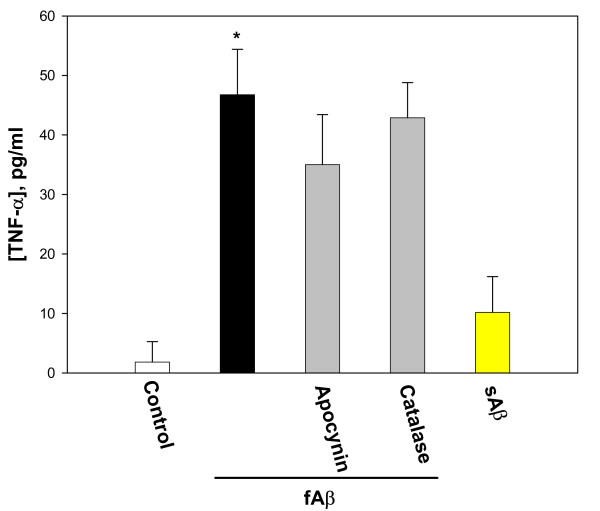
The early release of TNF-α by fibrillar Aβ_1–40_. Microglial cultures were incubated with 1 μM fibrillar (f) or soluble (s) Aβ_1–40_, alone of together with 10 μM apocynin or 60 IU/ml catalase for 1 hour. TNF-α levels were assessed in the media collected from the cultures. Data are expressed as mean of 4 experiments ± standard error; statistical analysis used: Student's t-test, p < 0.05; * – significant difference compared to the control.

**Figure 5:**
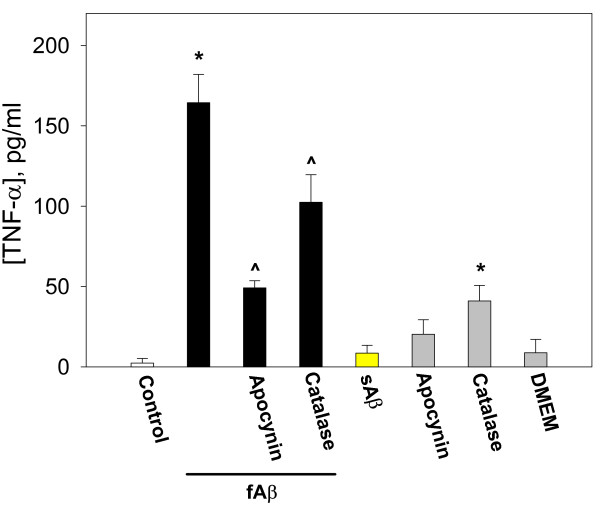
The effect of NADPH oxidase inhibitor and hydrogen peroxide scavengers on 24 hour treatment with Aβ_1–40 _peptide-induced TNF-α release from microglia. Microglial cultures were incubated with 1 μM fibrillar (f) or soluble (s) Aβ_1–40_, and/or with 10 μM apocynin, 60 IU/ml catalase, or 20 μM EUK-8/-134 for 24 hours. Then, TNF-α concentrations were measured in the cell incubation media. DMEM – microglial incubation medium not pre-incubated with cells. Data are expressed as mean of 6 experiments ± standard error; statistical analysis used: Student's t-test, p < 0.05; * – significant difference compared to the control; # – significant difference compared to samples treated with fibrillar Aβ only.

**Figure 6:**
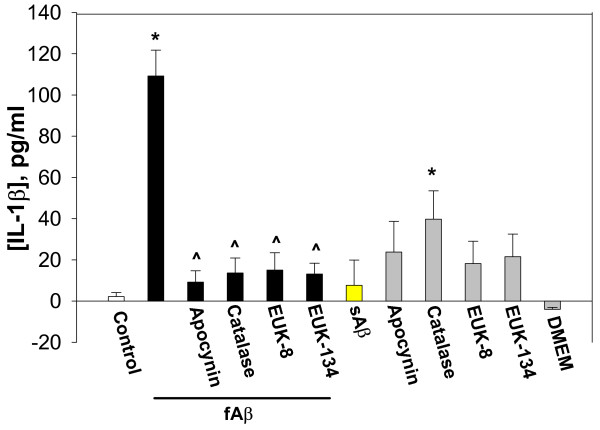
The effect of NADPH oxidase inhibitor and hydrogen peroxide scavengers on Aβ_1–40 _peptide-induced IL-1β release from microglia. Microglial cultures were incubated with 1 μM fibrillar (f) or soluble (s) Aβ_1–40_, and/or with 10 μM apocynin, 60 IU/ml catalase, or 20 μM EUK-8/-134 for 24 hours. Then, IL-1β concentrations were measured in the cell incubation media. DMEM – microglial incubation medium not pre-incubated with cells. Data are expressed as mean of 7 experiments ± standard error; statistical analysis used: Student's t-test, p < 0.05; * – significant difference compared to the control; # – significant difference compared to samples treated with fibrillar Aβ only.

**Figure 7:**
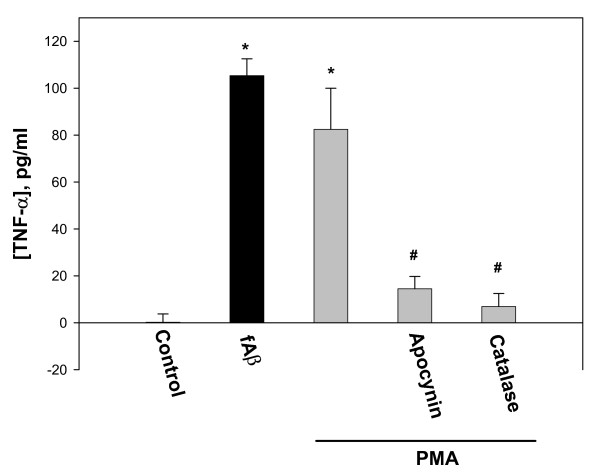
The effect of PMA on TNF-α release by microglia. Microglial cultures were incubated with 1 μM fibrillar (f) Aβ_1–40_, with 10 pg/ml of the NADPH oxidase activator phorbol 12-myristate 13-acetate (PMA), or with PMA plus either the NADPH oxidase inhibitor apocynin (10 μM) or the hydrogen peroxide converter catalase (60 IU/ml) for 24 hours. TNF-α concentrations were measured in the cell incubation media. Data are expressed as mean of 4 experiments ± standard error; statistical analysis used: Student's t-test, p < 0.05; * – significant difference compared to the control; # – significant difference compared to samples treated with PMA only.

**Figure 8:**
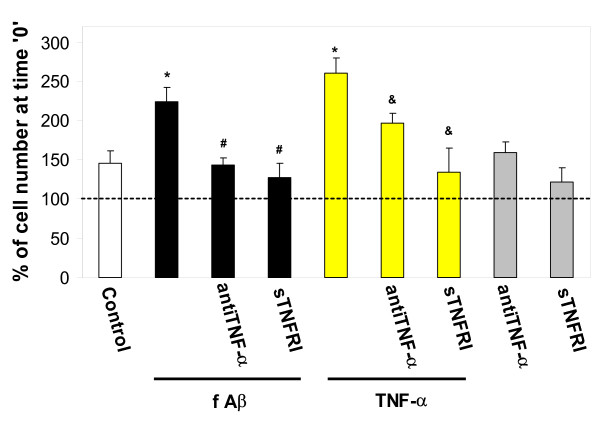
The effect of anti-TNF-α antibody on fibrillar Aβ_1–40_-induced microglial proliferation. Microglial cultures were incubated with 1 μM fibrillar (f) Aβ_1–40_, or fAβ together with either 40 μg/ml anti-rat TNF-α antibody or 10 ng/ml soluble TNF receptor inhibitor, or with 10 pg/ml TNF-α, alone or together with antibody or inhibitor, or with the antibody or the inhibitor alone for 24 hours. Cells were then counted and their numbers expressed as percentage of cell number at the start of the treatment, or time'0'. The dashed line indicates cell number at time '0'. sTNFRI – soluble TNF receptor inhibitor. Data are expressed as mean of 3–7 experiments ± standard error. Statistical analysis used: Student's t-test, p < 0.05; * – significant difference compared to the control; # – significant difference compared to samples treated with fibrillar Aβ_1–40 _only; & – significant difference compared to TNF-α only-treated samples.

### Detection of hydrogen peroxide

**Figure 9:**
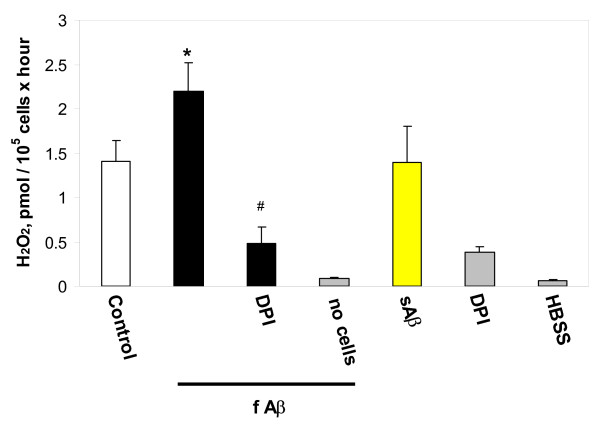
Production of hydrogen peroxide by isolated microglial cells in the presence of Aβ_1–40 _peptide. Microglial cells were incubated in HBSS with 1 μM fibrillar (f) or soluble (s) Aβ_1–40_, or with f Aβ_1–40 _together with 20 μM diphenylene iodonium (DPI) for 2 hours at 37°C. Hydrogen peroxide production was measured in a fluorometric assay. Data are expressed as mean of 4–7 experiments ± standard error. Statistical analysis used: Student's t-test, p < 0.05; * – significant difference compared to the control; # – significant difference compared to samples treated with fibrillar Aβ only.

Hydrogen peroxide formed by isolated microglia was measured in a fluorometric assay, using horseradish peroxidase oxidation of Amplex Red to fluorescent resorufin. The reaction mixture of a control sample contained 1 μM Amplex Red, 10 U/ml horseradish peroxidase and 3 × 10^5 ^microglia/ml resuspended in Hanks' balanced salt solution (HBSS; 5.33 mM KCl, 0.441 mM KH_2_PO_4_, 138 mM NaCl, 0.338 mM NaH_2_PO_4_, 4.17 mM NaHCO_3_, 1.26 mM CaCl_2_, 0.493 mM MgCl_2_·6H_2_O, MgSO_4_·7H_2_O, pH 7.4 at room temperature) with 50 mM freshly added glucose. Other samples had added either 1 μM fibrillar Aβ_1–40 _or 1 μM soluble Aβ_1–40_. Hydrogen peroxide levels in the samples were measured in a stirred cuvette using a Shimadzu RF-1501 spectrofluorophotometer (λ_ex _= 560 nm, λ_em _= 587 nm). Measurements were done immediately after sample preparation and repeated again after incubation for 2 hours at 37°C. The increase in fluorescence of each sample over two hours was converted to amount of hydrogen peroxide according to a calibration curve constructed using known concentrations of added hydrogen peroxide. The data (shown in Fig. 9) are presented as amount of hydrogen peroxide produced by 10^5 ^cells per 1 hour.

## Results

### The effect of Aβ_1–40 _on microglial proliferation

After incubation of pure microglial cultures with 1 μM fibrillar Aβ_1–40 _for 24 hours, the number of cells increased to 204 ± 9% of the cell number counted at time point '0' (Fig. 1), i.e. the cell density doubled in 24 hours. The number of cells in untreated control cultures did not significantly change over the same time period. When microglia were incubated with fibrillar Aβ_1–40 _for 48 hours, cells continued to proliferate to 372 ± 53% of the initial number, whereas in the untreated control the number of cells increased to 223 ± 28% of the initial number (Fig. 2). Soluble (non-fibrillized) Aβ_1–40 _used at the same concentration as the fibrillar peptide had no effect on cell proliferation rate, measured at 24 (Fig. 1) or 48 hours (Fig. 2).

We have recently found that microglial proliferation can be regulated by hydrogen peroxide derived from NADPH oxidase [10]. To test whether NADPH oxidase is involved in Aβ_1–40_-stimulated proliferation of microglia we incubated pure microglial cultures with fibrillar Aβ_1–40 _in the presence of 10 μM apocynin, an inhibitor of NADPH oxidase. Apocynin completely blocked the effect of Aβ_1–40 _in both 24- and 48-hour treatments (Fig. 1 &2). The hydrogen peroxide-degrading enzyme catalase (60 IU/ml), and its mimetics EUK-8 and EUK-134 (Mn-Salen compounds with both catalase and superoxide dismutase [18], both at 20 μM), also significantly decreased the effect of fibrillar Aβ_1–40 _(Fig. 1 &2) suggesting that hydrogen peroxide is important in the stimulation of microglial proliferation by the peptide. There was no effect of apocynin, catalase or EUKs on microglial proliferation without Aβ_1–40 _(Fig. 1 &2), and there was no increase in cell death caused by these compounds, as assessed by propidium iodide staining of the cultures (not shown).

Treatment of microglial cultures for 24 h with a low concentration of the NADPH oxidase activator PMA (10 pg/ml) caused an increase in proliferation rate similar to that induced by fibrillar Aβ over the same time period (compare Fig. 3 to Fig. 1), and this increase was completely prevented by apocynin and catalase. This suggests that activation of NADPH oxidase is sufficient to induce microglial proliferation via H_2_O_2 _production, and that activation of the oxidase by fibrillar Aβ could be sufficient to explain fibrillar Aβ-induced microglial proliferation.

### The effect of Aβ_1–40 _on inflammatory cytokine release by microglia

After incubation of pure microglial cultures with 1 μM fibrillar Aβ_1–40 _for 1, 6, 24 or 48 hours, media were collected from the cells and screened for TNF-α and/or IL-1β levels. We found that the medium TNF-α levels after 1-, 6-, 24- and 48-hour incubations with fibrillar Aβ were 47 ± 8, 186 ± 57, 164 ± 17 and 95 ± 7 pg/ml, respectively, whereas levels in the absence of Aβ were undetectable, undetectable, 22 ± 13 and 54 ± 14 pg/ml, respectively, after same time points. TNF-α levels in soluble Aβ-treated samples remained low: they were 10 ± 6, 12 ± 6, and 8 ± 5 pg/ml after 1, 6 and 24 hours, respectively.

There was no detectable IL-1β in fibrillar or soluble Aβ_1–40_-treated samples, or in untreated control samples, after 6 hours of incubation (data not shown). However, when cells were kept with the fibrillar peptide for 24 hours, IL-1β levels in the cell-conditioned medium increased to 109 ± 12 pg/ml, while IL-1β concentrations remained close to zero in controls and in samples treated with soluble Aβ (Fig. 6).

These experiments indicate that fibrillar Aβ_1–40 _peptide activates microglia to produce and/or release inflammatory cytokines. The release of TNF-α is much more rapid than that of IL-1β. Next, we tested whether this cytokine release is mediated by hydrogen peroxide from NADPH oxidase. Pure microglial cultures were incubated with Aβ_1–40 _peptide together with 10 μM apocynin, with 60 IU/ml catalase (for TNF-α and IL-1β measurements), or with 20 μM EUK-8 or EUK-134 (for IL-1β measurements), after which cytokine concentrations in the incubation media were assessed.

TNF-α levels in fibrillar Aβ-treated cultures were already elevated after 1 hour of treatment; it is therefore probable that Aβ is promoting release of pre-formed TNF-α, rather than (or in addition to) promoting TNF-α production *per se*. A 1-hour incubation with fibrillar Aβ peptide caused the medium TNF-α level to increase from 2 ± 3 to 47 ± 8 pg/ml, and neither apocynin nor catalase significantly inhibited this release (Fig. 4). A 1-hour treatment with soluble Aβ_1–40 _had no significant effect on the TNF-α concentration in the medium. These data suggest that early release of TNF-α from microglia in the presence of fibrillar Aβ_1–40 _occurs independently of NADPH oxidase activation and H_2_O_2 _formation.

After 24 hours of incubation with the NADPH oxidase inhibitor, apocynin, there was partial blockage of Aβ_1–40 _peptide-induced TNF-α release: cytokine levels in Aβ_1–40 _+ apocynin-treated samples were 70% lower compared to samples treated only with Aβ_1–40 _(Fig. 5). Catalase was also effective in decreasing (by 38%) the Aβ_1–40_-induced TNF-α release. However, none of the treatments completely prevented Aβ-induced increases in TNF-α levels after 24 hours. This suggests that the NADPH oxidase and H_2_O_2 _may mediate Aβ-induced TNF-α production, but not release.

Aβ_1–40_-induced IL-1β increases over 24 hours were almost completely stopped by apocynin, catalase and EUKs (Fig. 6), indicating that Aβ-induced production or release of IL-1β is dependent on hydrogen peroxide from active NADPH oxidase. Apocynin, catalase and EUKs alone also slightly increased IL-1β concentration in microglia-conditioned medium, but the increase was significant only with catalase treatment.

In order to test whether activation of NADPH oxidase would be sufficient to cause TNF-α production or release, we treated microglia with PMA (10 pg/ml) ± apocynin or ± catalase, and measured TNF-α in the medium after 24 hours. PMA did indeed increase TNF-α levels, to a degree similar to that caused by fibrillar Aβ, and this PMA-induced increase was blocked by apocynin and catalase (Fig. 7).

### The effect of TNF-α neutralisation on Aβ_1–40_-induced microglial proliferation

TNF-α is known to induce microglial proliferation, and we have previously shown that this induced proliferation is mediated by the NADPH oxidase [10]. The data presented elsewhere in this study suggest that in the presence of fibrillar Aβ_1–40_, TNF-α release may precede NADPH oxidase activation. Thus, TNF-α may mediate Aβ-induced microglial proliferation upstream of NADPH oxidase. To test this hypothesis, we incubated microglial cultures with 1 μM fibrillar Aβ_1–40 _and either 40 μg/ml anti-TNF-α monoclonal antibody or 10 ng/ml soluble TNF receptor inhibitor for 24 hours and assessed proliferation of the cells. Both anti-TNF-α antibody and soluble TNF receptor inhibitor completely inhibited the increase in proliferation induced by fibrillar Aβ (Fig. 8), indicating that Aβ-induced proliferation is mediated by TNF-α release. As expected, the antibody and the inhibitor also prevented microglial proliferation induced by TNF-α itself (Fig. 8).

### The effect of Aβ_1–40 _on hydrogen peroxide generation by microglia

The above data suggest that fibrillar Aβ peptide stimulates microglia in part via activating hydrogen peroxide production from the microglial NADPH oxidase. We therefore measured hydrogen peroxide production by microglia in the presence and absence of fibrillar Aβ_1–40_. There was no detectable change in the rate of hydrogen peroxide production immediately after addition of the peptide (1 μM), even when the Aβ concentration was increased up to 50 μM (data not shown). However, after 2 hours of incubation with 1 μM fibrillar Aβ_1–40_, microglia produced significantly larger amounts of hydrogen peroxide than did untreated control cells (Fig. 9). Cells that were incubated with the soluble form of the peptide produced the same amount of hydrogen peroxide as the untreated controls. An inhibitor of NADPH oxidase, diphenylene iodonium (DPI, 20 μM), prevented hydrogen peroxide generation by fibrillar Aβ_1–40 _peptide-treated, as well as by untreated cells. Hydrogen peroxide production by the incubation medium alone (0.17 pmol/ml hour) or by 1 μM fibrillar peptide incubated in the medium without cells (0.25 pmol/ml hour) was low compared to that in the presence of cells (4.2 pmol/ml hour in absence of Aβ, 6.6 pmol/ml hour in the presence of fibrillar Aβ with 300,000 cells/ml).

In this study we found that neutralisation of TNF-α blocks fibrillar Aβ-induced microglial proliferation (Fig. 8). We have reported previously that TNF-α stimulates microglial proliferation, activating NADPH oxidase-derived hydrogen peroxide production [10]. Taken together, these data suggest that fibrillar Aβ-induced increases in hydrogen peroxide production by microglia can be mediated by TNF-α. In contrast, soluble TNF receptor inhibitor (100 ng/ml) was not able to prevent fibrillar Aβ-caused stimulation of hydrogen peroxide production over 2 hours, but did effectively eliminate a 100 pg/ml TNF-α-induced increase in hydrogen peroxide formation (data not shown). This suggests that Aβ-induced activation of NADPH oxidase is not mediated by TNF-α in this time period.

## Discussion

Beta amyloid has previously been reported to stimulate superoxide or hydrogen peroxide production from isolated microglia via activation of NADPH oxidase [9,16,17], and our results are consistent with this. As we have previously reported that hydrogen peroxide from PHOX stimulates microglial proliferation [10], and others have reported that hydrogen peroxide from PHOX stimulates microglial cytokine production [11], we tested whether Aβ_1–40 _could stimulate microglial proliferation and cytokine production via activating hydrogen peroxide production from PHOX. Fibrillar Aβ_1–40 _did indeed stimulate the proliferation of isolated microglia, measured at both 24 and 48 hours after Aβ_1–40 _addition, whereas non-fibrillized Aβ_1–40 _had no effect on the rate of proliferation (Fig. 1 &2). The stimulation of proliferation induced by fibrillar Aβ_1–40 _was completely prevented by either a specific inhibitor of PHOX (apocynin) or agents that remove hydrogen peroxide (catalase, EUK-8, EUK-134), implicating hydrogen peroxide from PHOX as the mediator of Aβ_1–40_-induced proliferation.

Microglial proliferation is associated with neuronal damage in a variety of pathologies such as ischemia [23] or compression injury [24], as well as in different animal models of Alzheimer's disease [25,26]. Hydrogen peroxide and the NADPH oxidase can stimulate proliferation in a number of different cell types [27-29]. It has been demonstrated that hydrogen peroxide inhibits CD45 (a transmembrane tyrosine phosphatase expressed in cells of monocytic lineage) [30], and it has recently been shown that the activation of CD45 blocks GM-CSF-induced microglial proliferation [31]. There is evidence that hydrogen peroxide can oxidise critical sulphydryl groups in tyrosine phosphatases [32], which results in increased tyrosine phosphorylation and prolongation of mitogenic signalling [31,33]. Thus CD45 might be one potential target for hydrogen peroxide in regulating microglial proliferation.

**Figure 10:**
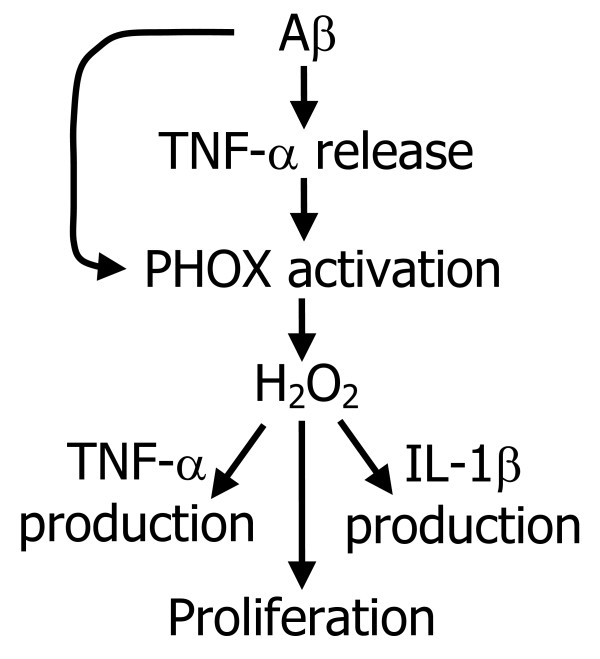
A suggested model for Aβ-induced microglial proliferation and activation.

Proliferation of microglia is a key component of the brain's inflammatory response, as microglia are central to this response and levels of microglia in the resting (non-inflammed) brain are low (roughly 5% of all brain cells) [34]. Microglia are a major source of pro-inflammatory cytokines, particularly IL-1β and TNF-α, that cause inflammatory activation of the brain. We found that Aβ_1–40 _induces IL-1β production by isolated microglia, and that this induced production is almost completely blocked by either a specific inhibitor of PHOX (apocynin) or agents that remove hydrogen peroxide (catalase, EUK-8, EUK-134, Fig. 6), implicating hydrogen peroxide from PHOX as the mediator of Aβ_1–40_-induced IL-1β production. Fibrillar Aβ_1–40 _also induced TNF-α production and/or release from microglia (Fig. 4 &5). The Aβ_1–40_-induced TNF-α production and/or release was much more rapid than the Aβ_1–40_-induced IL-1β production and/or release, was only partially sensitive to catalase and apocynin at 24 hours, and was insensitive at 1 hour (Fig. 4 &5). This suggests that Aβ_1–40 _causes early TNF-α release by mechanisms unrelated to NADPH oxidase activation, and presumably not mediated by gene expression and translation, whereas the later TNF-α production may be mediated by PHOX/H_2_O_2 _regulated gene transcription and translation. As Aβ causes early TNF-α release (Fig. 4), and TNF-α stimulates microglial proliferation (Fig. 8), we tested whether Aβ_1–40_-induced microglial proliferation might be mediated by TNF-α release. We found that an anti-TNF-α antibody and a soluble TNF receptor inhibitor both prevented Aβ-induced proliferation (Fig. 8), indicating that Aβ-induced proliferation is mediated by TNF-α release. As TNF-α induces microglial proliferation via stimulating the NADPH oxidase to produce H_2_O_2 _[10], this is consistent with Aβ-induced proliferation being mediated by PHOX. A minimal model consistent with these results is presented in Fig. 10.

The concentration of Aβ_1–40 _used in this work (1 μM) is a pathophysiologically relevant concentration that has little direct toxicity to neurons or astrocytes, but can cause toxicity to neurons indirectly by inflammatory activation of co-cultured microglia [9]. Aβ_1–40 _peptide is more abundant in the brain than Aβ_1–42 _peptide [35]. The means by which these peptides, when fibrillized, activate NADPH oxidase in microglia is unclear; it may involve stimulation of phagocytosis via a receptor complex that includes a β1 integrin [36], or it may be mediated by TNF-α release. Whatever the mechanism, these data suggest that TNF-α and NADPH oxidase may be potential targets, and that apocynin and the EUKs may be potential drugs for Alzheimer's-associated inflammation and inflammatory neurodegeneration.

## Conclusion

We found that fibrillar Aβ-induced microglial proliferation is mediated by NADPH oxidase, hydrogen peroxide and TNF-α. In the presence of Aβ_1–40_, microglial cells prolife rate, and this is prevented either by inhibiting NADPH oxidase, by removing hydrogen peroxide, or by neutralising released TNF-α with an antibody.

Fibrillar Aβ_1–40 _induces a rapid, NADPH oxidase-independent release of TNF-α by microglial cultures; however, prolonged exposure to the peptide causes PHOX-dependent TNF-α and IL-1β production that is prevented by inhibiting NADPH oxidase or by removing hydrogen peroxide.

## Competing interests

The author(s) declare that they have no competing interests.

## Authors' contributions

AJ carried out all the experiments presented in the paper, created Figures and wrote Methods and Results. PKM did many preliminary studies, taught AJ how to prepare glial cultures as well as other assays used for this study, and made helpful comments preparing the manuscript. AT synthesized the Aβ_1–40 _peptide. MS synthesized EUK-8 and EUK-134 and helped to write the manuscript providing useful suggestions. GCB conceived of the study, and participated in its design and coordination, and wrote Introduction and Discussion of the manuscript. All the authors have read and approved the final manuscript.
